# Planting Systems Affect Soil Microbial Communities and Enzymes Activities Differentially under Drought and Phosphorus Addition

**DOI:** 10.3390/plants11030319

**Published:** 2022-01-25

**Authors:** Olusanya Abiodun Olatunji, Kaiwen Pan, Akash Tariq, Gideon Olarewaju Okunlola, Dong Wang, Idris Olawale Raimi, Lin Zhang

**Affiliations:** 1 Department of Plant Biology, Faculty of Basic and Applied Sciences, Osun State University, Osogbo 230261, Nigeria; gideon.okunlola@uniosun.edu.ng; 2 CAS Key Laboratory of Mountain Ecological Restoration and Bioresource Utilization & Ecological Restoration and Biodiversity Conservation Key Laboratory of Sichuan Province, Chengdu Institute of Biology, Chinese Academy of Sciences, Chengdu 610041, China; zhanglin@cib.ac.cn; 3 Xinjiang Key Laboratory of Desert Plant Roots Ecology and Vegetation Restoration, Xinjiang Institute of Ecology and Geography, Chinese Academy of Sciences, Urumqi 830011, China; akash.malik786@mails.ucas.ac.cn; 4 State Key Laboratory of Desert and Oasis Ecology, Xinjiang Institute of Ecology and Geography, Chinese Academy of Sciences, Urumqi 830011, China; 5 College of Environmental Science and Engineering, China West Normal University, Nanchong 637000, China; 459289120@163.com; 6 Department of Biology, Sefako Makgatho Health Sciences University, P.O. Box 139, Medunsa 0204, South Africa; 201710318@swave.smu.ac.za

**Keywords:** drought, ecosystem functions, microbial function, phosphorus, monoculture, mixed culture

## Abstract

The use of phosphorus (P) to alleviate soil nutrient deficiency alters resources in plant and microbial communities, but it remains unknown how mixed and monospecific planting of forest tree species shape soil microbial structure and functions in response to drought and its interplay with phosphorus addition. We investigated the microbial structure and chemical properties of forest soils planted with *P. zhennan* monoculture, *A*. *cremastogyne* monoculture, and their mixed cultures. The three planting systems were exposed to drought (30–35% water reduction) and the combination of drought with P. A well-watered treatment (80–85% water addition) of similar combinations was used as the control. Planting systems shaped the effects of drought on the soil microbial properties leading to an increase in nitrate nitrogen, urease activity, and microbial biomass carbon in the monocultures, but decrease in mixed cultures. In the monoculture of *P. zhennan*, addition of P to drought-treated soil increased enzyme activities, the concentration of dissolved organic nitrogen, and carbon, leading to increase in the total bacteria, G^+^ bacteria, and arbuscular mycorrhizal fungi. Except in the drought with P addition treatment, the impact of admixing on total phospholipid fatty acids (PLFAs), bacterial PLFA, and fungi PLFA was synergistic in all treatments. Our findings indicated that in monoculture of *P. zhennan* and its mixed planting with *A. cremastogyne*, greater biological activities could be established under drought conditions with the addition of P.

## 1. Introduction

Anthropogenic global change, characterized by diminished precipitation regimes and nutrient enrichment, among other factors, is a phenomenon that will influence terrestrial ecosystems functions worldwide [1,2]. Drought in the soil environment could affect microbial communities and enzyme activities, which are essential components in the biogeochemical processes. Moreso, reductions in water availability could limit or aggravate the effects of phosphorus deposition by enhancing its immobility in soil [3]. The use of fertilizers such as phosphorus (P) could decrease or increase the microbial biomass and may also exert selective forces on microbial groups by favoring some over the other, such as bacteria over fungi [3,4,5]. Dong et al. [4] and Lie et al. [5] reported that phosphorus addition increased total phospholipid fatty acid (PLFAs), bacterial PLFA, and fungal PLFA, Drought could induce osmotic stress on the plant, which might be accompanied by a trade-off between plant and soil, resulting in plants developing a range of adaptation strategies. The adaptation strategy used by the plant may lead to disparity in soil properties (e.g., carbon and nitrogen pools, nitrogen mineralization rate). The difference in soil chemical properties may be more complex when monospecific species with different requirements for water and nutrients are planted in a mixed culture [6,7]. The type of planting practice may result in a turn in the alteration of the community composition of the soil microbe, enzyme activities, and ecosystem functions [8]. However, the underlying impacts of mixed cultivation of different monospecific tree species under drought and P addition on soil microbial biomass and community composition, along with the activities of enzymes engaged in nitrogen, carbon, and phosphorus cycling, are mostly unknown.

As depicted by niche complementarity principle [9], a planting system of mixed species may serve as an alternative to lessen drought effect on plants, as responses to environmental conditions are likely to vary in a mixed system that comprises species with diverse functional traits and tactics for resource utilization [10]. However, it is not yet known whether the same scenarios are applicable to soil microbial community structure in drought events. A planting system of mixed stands with subtle variations in belowground functional traits may lead to two possible effects on the soil microbial characteristics, these main effects being additive or nonadditive (synergistic or antagonistic), with hypothetically broad consequences for soil biochemical properties and stability [11,12,13]. Because the potential effects of plant species can be altered within days by the slightest change in soil water content and nutrient addition, differences in tree species mixtures might be anticipated to also influence composition of soil microbial community along with the enzyme activities [4,7,9]. Nonetheless, Orwin and Wardle [14], in a study of three common pasture plants, reported no consequence of species mixtures on microbial responses to reduced soil water content. In contrast, Sun et al. [10] observed that a mixed culture of two different plant species enhanced the microbial community’s structural ability to return to control levels after extreme rainfall effects. Moreover, microbial community structure is a multifaceted “black box” that needs a multiconceptual tactic [15], and knowledge of the possible interplay within complex soil microbial communities and how they recover or respond to disturbances such as climate extremes, remains relatively limited. Therefore, it is reasonable to conduct investigations of the concurrent effects of drought and increased level of P addition under different planting systems, because such an investigation could provide critical insights into how microbial community structures respond in the context of monospecific and mixed plant species management under climate change.

In line with the recent call to embrace multiple approaches in examining single question [16], we studied the responses of soil microbial communities to multiple factors. These factors include P addition, drought, and the additive or nonadditive consequences of species mixtures using *Phoebe zhennan* and *Alnus cremastogyne* saplings as a model system. *Phoebe zhennan* and *A. cremastogyne* were chosen because their cultivation has gained more attention in conservation and ecological restoration projects [17]. Notably, they could serve as a prototype to understand the nature of ecosystem functions of single and mixed-species plantations under climate change. We hypothesize that (1) the sensitivity to drought by the microbial community along with the enzyme activities will be more pronounced under monoculture than in the mixed culture, (2) disparities in microbial community composition will be mediated via phosphorus addition under drought conditions, and this effect will be stronger in mixed cultures than in monocultures, and (3) changes in biomass and microbial community will be aligned with the changes to soil chemical properties mediated by the planting system under a climate change scenario.

## 2. Results

### 2.1. Soil Chemical Parameters

Drought increased the concentrations of dissolved organic carbon (DOC) in PZ monoculture but decreased it in AC monoculture (Table 1). Drought significantly increased NO_3_^−^–N and aP in the PZ and AC monocultures (*p* < 0.05). Compared to the controls, drought increased dissolved organic nitrogen (DON) concentration of all the planting systems (*p* < 0.05) (Table 1). Interplay of planting system with water treatments and of planting system with P influenced aP, DON, and DOC (Table S1). Drought and P together increased the concentrations of DOC and DON in PZ (*p* < 0.05) (Table 1). Overall, P addition to drought-treated soil significantly increased the concentration of aP in all three planting systems (Table 1).

### 2.2. Soil Microbial Biomass

**Figure 1:**
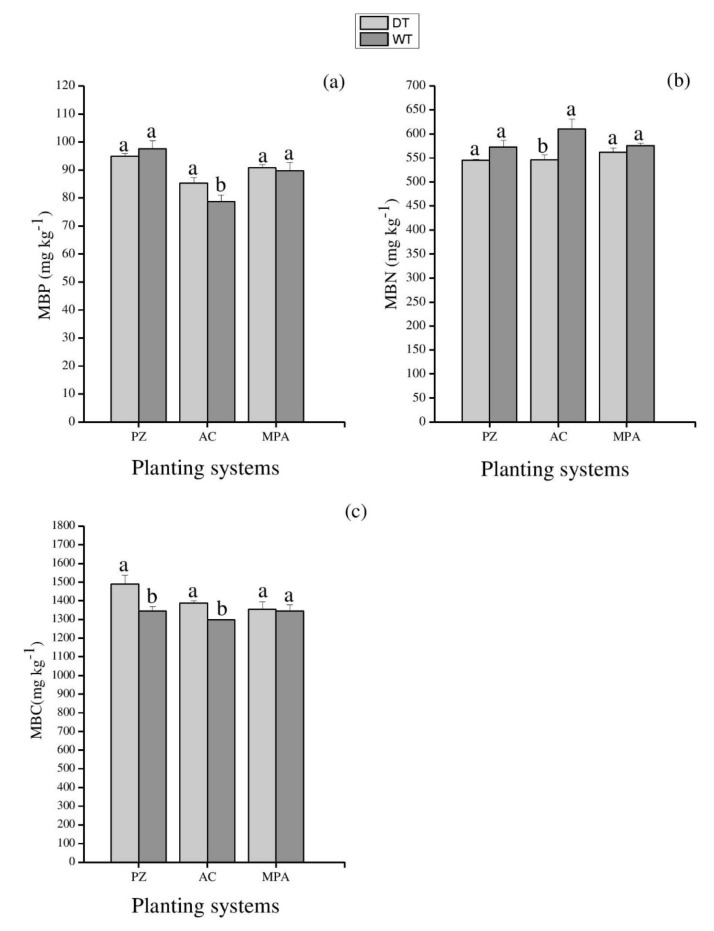
The drought effects on soil microbial biomass under different plantings systems. (**a**) MBP: microbial biomass phosphorus; (**b**) MBN: microbial biomass nitrogen, and (**c**) MBC: microbial biomass carbon. DT: drought; WT: well-watered. PZ: *Phoebe zhennan;* AC: *Alnus cremastogyne*; MPA: mixed culture of *Phoebe zhennan* and *Alnus* *cremastogyne*. Different lowercase letters indicate significant difference between the treatments. Bar shows mean ± standard error (SE). Significant at *p* < 0.05.

**Figure 2:**
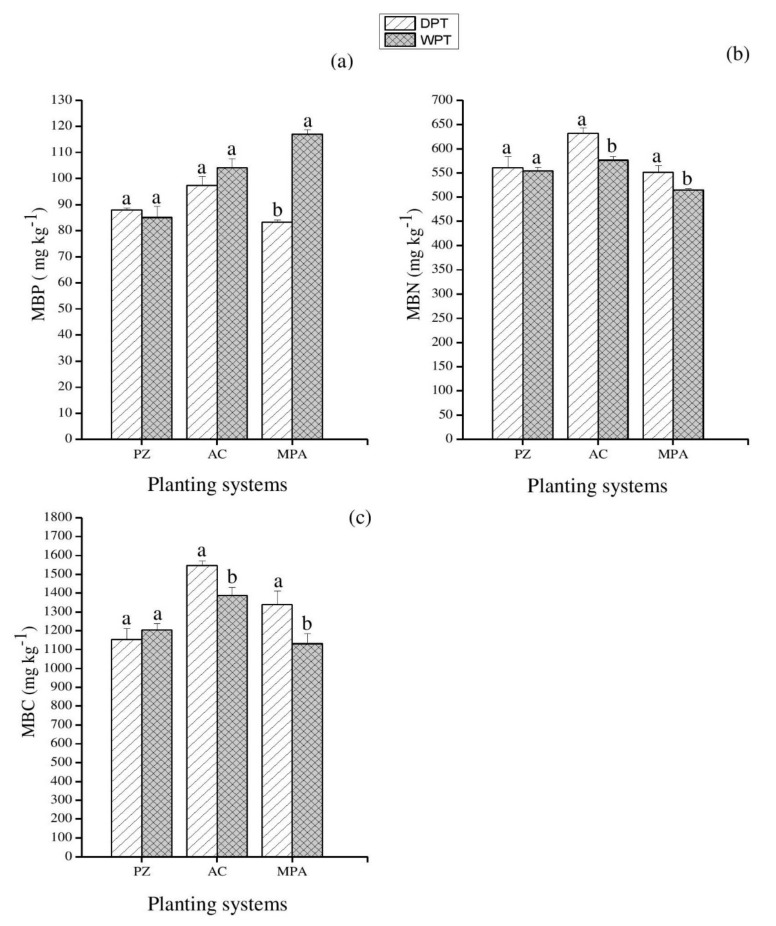
The drought with P addition effects on soil microbial biomass under different plantings systems. (**a**) MBP: microbial biomass phosphorus; (**b**) MBN: microbial biomass nitrogen, and (**c**) MBC: microbial biomass carbon. DPT: drought with phosphorus addition; WPT: well-watered with phosphorus addition. PZ: *Phoebe zhennan;* AC: *Alnus cremastogyne*; MPA: mixed culture of *Phoebe zhennan* and *Alnus cremastogyne*. Different lowercase letters indicate significant differences between the treatments. Bar shows mean ± standard error (SE). Significant at *p* < 0.05.

The microbial biomass was markedly influenced by planting systems, water treatments, and P addition (Table S2). Drought increased microbial biomass phosphorus in the AC system (*p* < 0.05) (Figure 1a). MBC was significantly higher in PZ and AC systems when subjected to induced drought treatments (*p* < 0.05) (Figure 1c). Overall, drought decreased the MBN in all the planting systems but significantly reduced it only in the AC system when compared to the controls (well-watered treatments) (Figure 1b). Compared to the control treatment (well-watered + P addition), the MBN and MBC significantly increased by drought together with P treatment in the AC and MPA planting systems (*p* < 0.05) (Figure 2b,c). There were no significant effects of the DPT on the microbial biomass in PZ systems.

### 2.3. Soil Microbial Community

**Figure 3:**
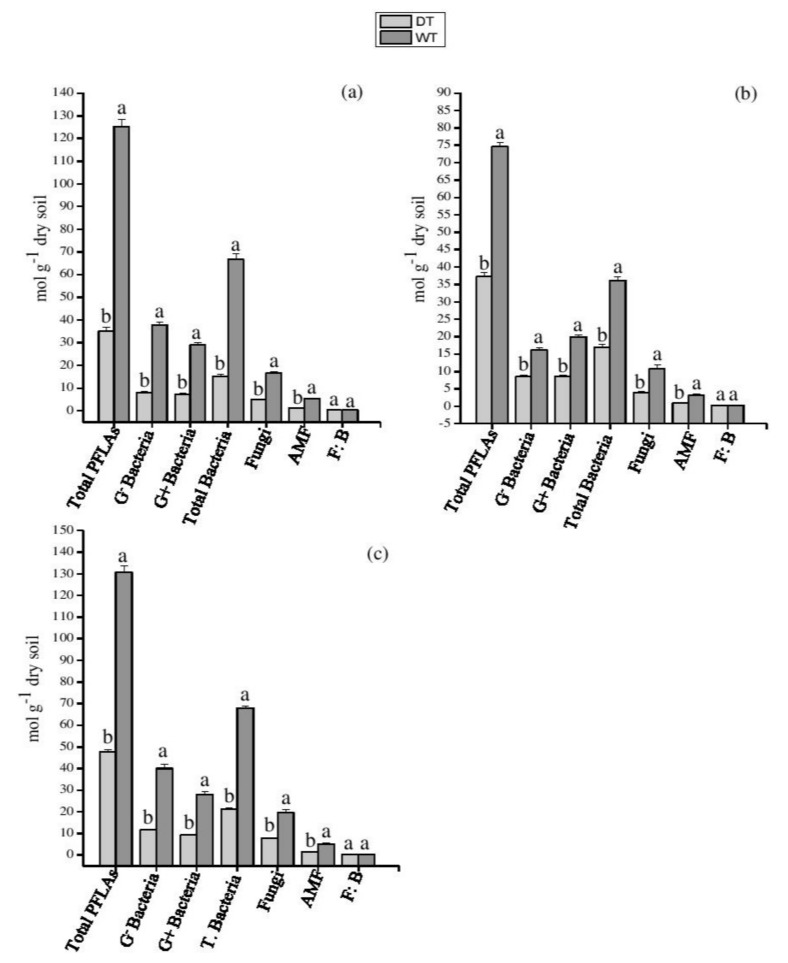
Impact of drought on soil microbial community composition under different plantings systems. (**a**) PZ: *Phoebe zhennan;* (**b**) AC: *Alnus cremastogyne,* and (**c**) MPA: mixed culture of *Phoebe zhennan* and *Alnus cremastogyne*. DT: drought; WT: well-watered. Different lowercase letters indicate significant differences between the treatments. Bar shows mean ± standard error (SE). Significant at *p* < 0.05.

**Figure 4:**
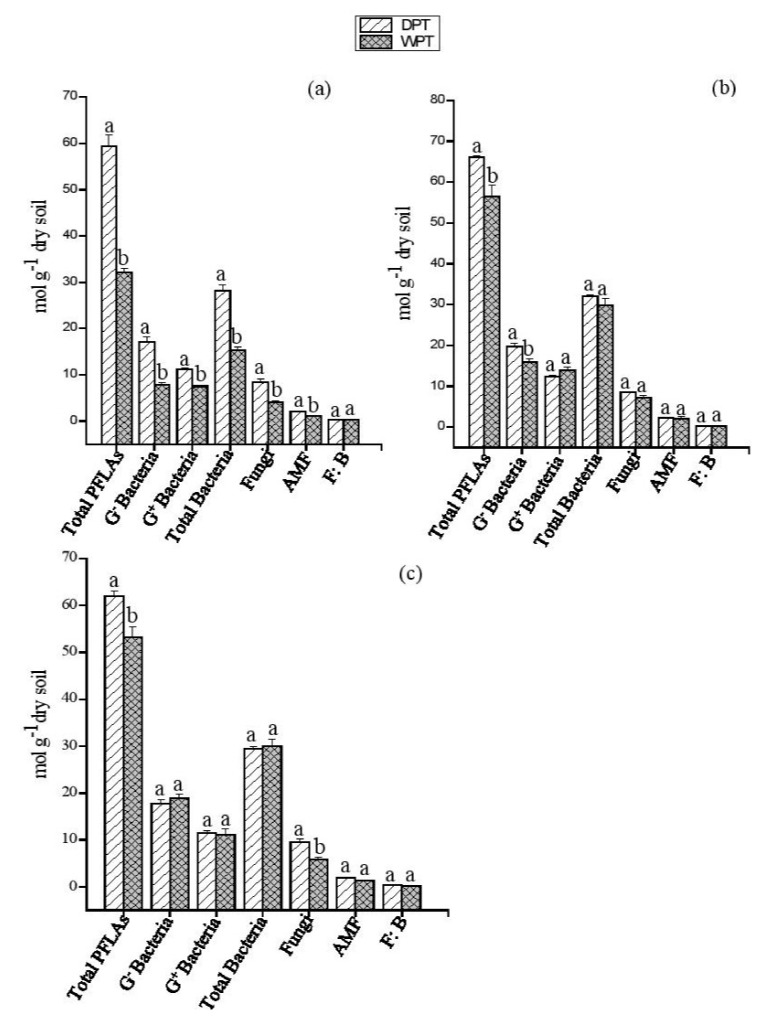
Impact of drought with P addition on soil microbial community composition under different plantings systems. (**a**) PZ: *Phoebe zhennan;* (**b**) AC: *Alnus cremastogyne,* and (**c**) MPA: mixed culture of *Phoebe zhennan* and *Alnus cremastogyne*. DPT: drought with phosphorus addition; WPT: well-watered with phosphorus addition. Different lowercase letters indicate significant differences between the treatments. Bar shows mean ± standard error (SE). Significant at *p* < 0.05.

The tPLFAs, total bacteria, G^−^, and G^+^ bacteria were affected by the interplay of water with planting systems (Table S3). Drought decreased all the microbial groups in each planting system (*p* < 0.05), except fungi bacterial ratio (F/B) (Figure 3). The interplay of P and water treatments exert significant impact on microbial communities (Table S3), which varied with the planting system (Figure 4). P addition under drought condition significantly increased G^−^ bacteria in PZ and AC systems (*p* < 0.05) (Figure 4a,b). In the PZ system, the fungi, total bacteria, G^+^ bacteria, and AMF were significantly increased by P when added to the soil under drought treatment (Figure 4a); however, in AC and MPA systems, there were no significant differences compared to their control treatments (well-watered + P addition) (Figure 4b,c). Overall, the addition P under drought condition increased the total PLFAs in all the planting systems (*p* < 0.05), and significantly increased the fungi PLFAs in the PZ and MPA systems (Figure 4).

### 2.4. Soil Enzyme Activities

Induced drought decreased alkaline phosphate in PZ and AC systems and increased (*p* < 0.05) urease activity when compared to the control (well-watered treatment) (Table 2). Drought increased alkaline phosphate activity in the MPA system (*p* < 0.05), but reduced urease activity. β- glucosidase activity was decreased by drought treatments in all the planting systems (Table 2). Except for β-glucosidase, enzyme activities were significantly influenced by the addition of P together with water treatments, (Table S4). The supply of P to drought-treated soil improved all enzyme activities in the PZ system, but only increased β-glucosidase and urease activities in the MPA system. The alkaline phosphate, β-glucosidase, and urease in AC system under drought with P addition treatment were equal to or significantly lower than those under the well-watered with P addition treatment (Table 2).

### 2.5. Nonadditive and Additive Effects

**Figure 5:**
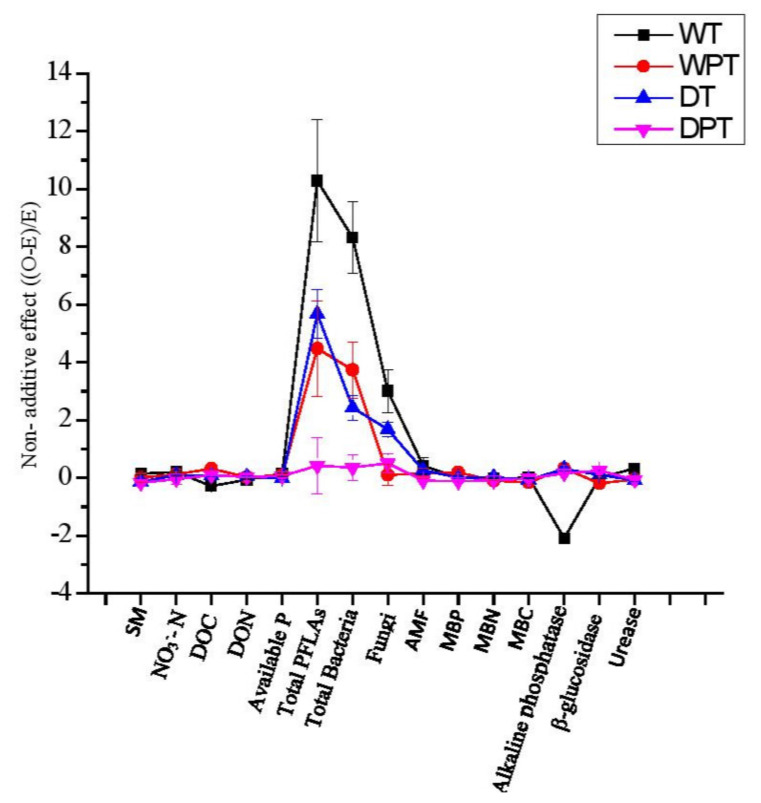
Nonadditive effects of species mixture on soil chemical properties, microbial community, and enzymes activities. D: drought; W: well-watered; DP: drought with phosphorus addition; WP: well-water with phosphorus addition. SM: soil moisture (%); DOC: dissolved organic carbon (mg/kg); DON: dissolved organic nitrogen (mg/kg); NO_3_^−^–N: soil nitrate nitrogen (mg/g); aP: available phosphorus (mg P kg^−1^ soil); AMF: arbuscular mycorrhizal fungi. Significant *p*-values (*p* < 0.05). Values differ significantly from zero indicate nonadditive, while the values that are not significantly departed from zero indicate additive mixing effects. Positive strong NAE values departed from zero indicate synergistic mixing effects while negative values departed from zero indicate antagonistic mixing effects.

The strength of the nonadditive impacts of tree mixtures was diverse with water treatments, P addition, and their interaction (Figure 5). There was antagonistic nonadditive effect of species mixture on alkaline phosphate activities in well-watered treatments. Strong synergistic impacts of species mixture were found for total PLFAs, bacteria, and fungi in all treatments, except in the drought with P addition (Figure 5).

### 2.6. Links among Soil Enzyme Activities, Soil Chemical Variables, and Microbial PLFAs

**Figure 6:**
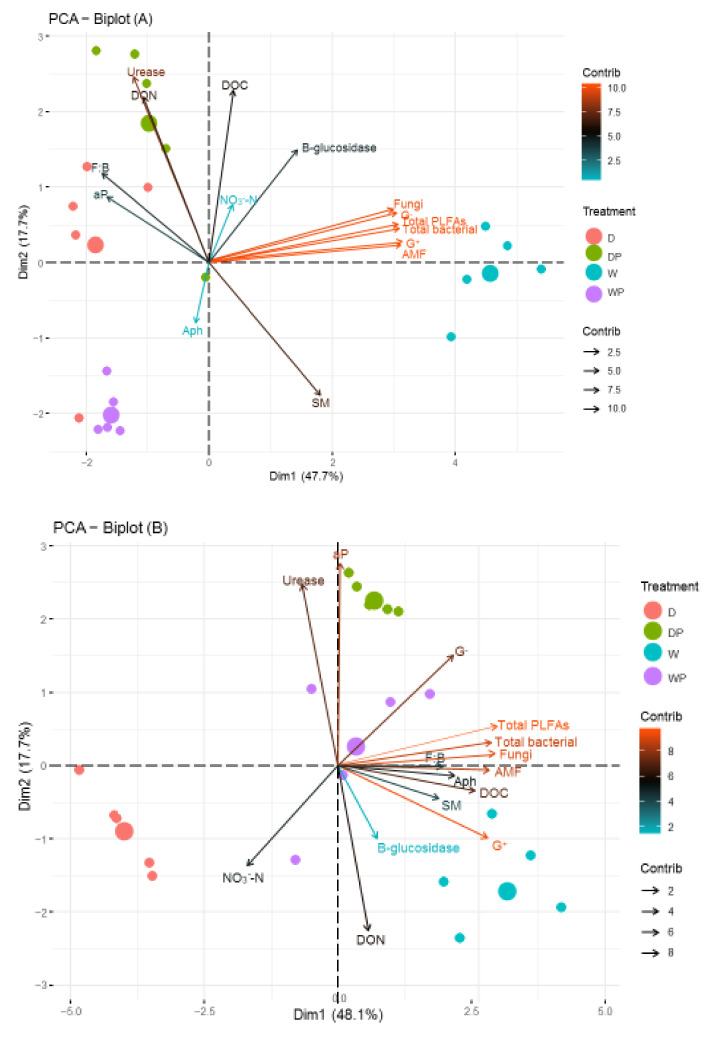

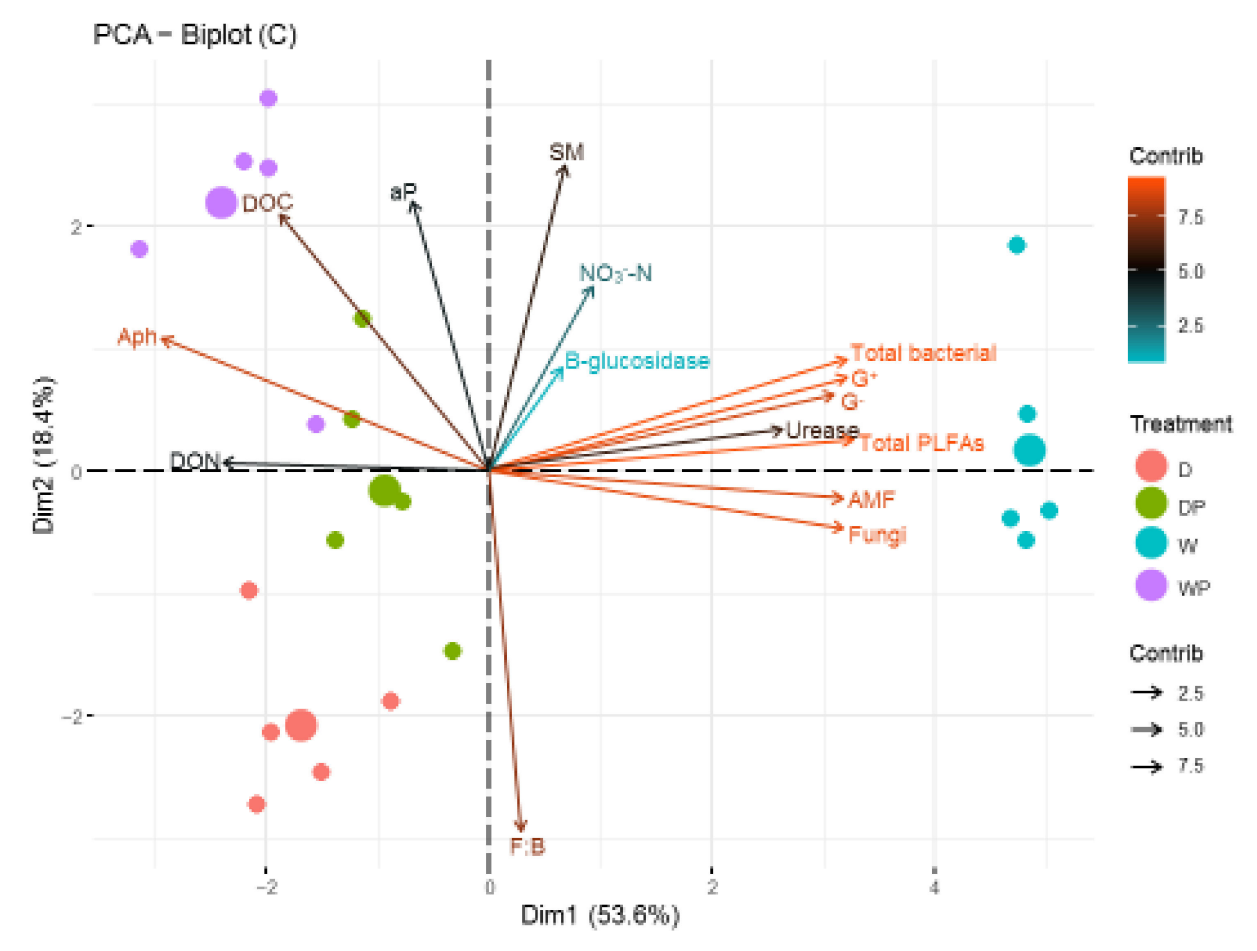
Principal component analysis of the soil variables under (**A**) monoculture of *P. zhennan*; (**B**) *Alnus cremastogyne,* and (**C**) mixed culture of *Phoebe zhennan* and *Alnus cremastogyne.* The length of the arrow line of the variables is plotted as the strength of contribution to community structure. SM, soil moisture (%); DOC, dissolved organic carbon (mg/kg); DON, dissolved organic nitrogen (mg/kg); NO_3_^−^–N, soil nitrate nitrogen (mg/g); aP, available phosphorus (mg P kg^−1^ soil); AMF, arbuscular mycorrhizal fungi; G^+^ bacteria, and G^−^ bacteria; aph, alkaline phosphate; BG, *β-*glucosidase.

The first component of the PCA explains 47.7%, 48.1%, and 53.6% of the disparity in microbial community structure of PZ, AC, and MPA, respectively (Figure 6). The second component explains 17.7%, 17.7%, and 18.4% under PZ, AC, and MPA, respectively. Distinct treatments effects were observed on the microbial community under each planting system (Figure 6). The total PLFAs, total bacteria, G^+^ bacteria, G^−^ bacteria, fungi PLFAs, and AMF were significantly positively associated with the soil moisture (SM) and *β-*glucosidase activities in the PZ system (Figure 6A). The microbial community in AC correlated significantly positively with soil moisture, DOC, and alkaline phosphate activities, but correlated negatively with NO_3_^−^–N (Figure 6B). Except for F/B in MPA system, the microbial community was significantly negatively related (*p* < 0.01) with DON and alkaline phosphate activities, but positively correlated with urease activities (Figure 6C).

## 3. Discussion

### 3.1. Drought Effects on the Soil Microbial Community Structures among Planting System

This study found that drought stress induced by reduction in soil water content had significant negative impact on the biomass and community composition of the soil microbe, enzyme activities, and soil chemical properties. Despite that microbial biomass is thought to upsurge in all cases when soil is vegetated [18,19,20], the microbial biomass C in this study demonstrated a dynamic response to drought by increasing in the monocultures but no significant response in the mixed planting systems, whereas drought decreased microbial N in all three planting systems. This observation highlighted that although drought increased concentration of some soil chemical variables, the increase varies with planting systems and does not automatically result in an increase in microbial biomass and community composition. Indeed, drought increased the DOC in the monoculture of *P. zhennan* and in the mixed culture with *A.* *cremastogyne* but increased the microbial biomass C in monoculture of *P. zhennan* and not in the mixed culture. Despite the increase in the NO_3_^−^–N and aP in the monocultures and an increase in DON in all planting systems under the drought condition, the deleterious effect of drought on total PLFAs, G^−^-and-G^+^ bacteria, total bacteria, fungi, and AMF was confirmed in all planting systems. This suggested that drought abridged root growth and nutrient uptake regardless of the planting system, and possibly prompted the death of weak resistant microbial populations, as substrates from the roots were to improve microbial activity [21]. Further, our findings from the PCA analysis of the microbial community indicated that moisture reduction exceeds the range of tolerance for microbial community composition regardless of planting system.

Deviation occurred from the postulation of microbial resource distribution, which stated that microbes are likely to reallocate their resource from synthesizing nutrient-acquiring enzymes once a nutrient remains readily accessible [22]. The present findings indicated that, under drought event, enzyme activities do not unceasingly increase or decrease despite nutrient decreases or increases. Indeed, in line with highlights of Dong et al. [23], higher urease activity in the monocultures of *P. zhennan* and *A. cremastogyne* under the drought condition were concomitant with higher NO_3_^−^–N and DON, whereas, in contrast, alkaline phosphate declined. Similar to the suggestion of Fang et al. [24], while drought increased alkaline phosphate activity in the mixed cultures of *P. zhennan* and *A. cremastogyne,* it decreased the available soil phosphorus. Therefore, the increase and decrease of soil enzymes involved in the P, N, and C cycles in both monoculture and mixed cultures under the drought condition suggests that planting systems may play a legacy role in regulating the activities of the soil enzymes and supply of nutrients under drought [23,25,26]. Overall, our findings affirm the negative consequences of drought on soil community, with outcome on the microbial functions.

### 3.2. Differential Response of Soil Microbial Community Structure to the Combined Drought with P Treatment

Previous studies on climate change have shown that alterations to, as well as relations amongst, precipitation, microclimate, soil, and root exudates, could control the composition as well as the function of microbial communities and enzyme activities [27,28]. In this study, drought interaction with P addition changed the pattern of responses of soil chemical properties, enzymes, biomass, and microbial community structure. The PCA analysis showed a clear separation of DP treatment from WP. The combination of P and drought wielded a positive impact on microbial biomass C and N of the *A. cremastogyne* monoculture and also its mixture with *P. zhennan*, but these effects were absent in the monoculture of *P. zhennan.*

Interestingly, the supplementation of the drought-treated soil with P resulted in a greater abundance of microbial PLFAs in *P. zhennan* monoculture [29]. The mechanism behind the upsurge in the microbial community of *P. zhennan* could be credited to an increase in amount of P supplied and dissolved organic carbon, which signify labile C and serve as primary source of energy for microbes [30], and an increase in DON (Table 1). The increase in DOC and DON could be attributed to reduction in nutrient uptake of *P. zhennan*. Moreover, *P. zhennan* is a slow-growing forest tree with a low mineral absorption rate, hence the availability of more resource for microbial use. Further, the observed higher level of enzyme activities in the monoculture of *P. zhennan* was inconsistent with previous studies [22,24,31]. Meanwhile, the results are consistent with the highlight of Dong et al. [4], that a higher level of enzyme activities could be attributed to higher organic C and N content, which enhanced microbial activities. This study reveal that (i) enzymes were easily stimulated by substrates, and addition of P might be crucial to the nutrient transformation of *P. zhennan* during drought conditions; (ii) under drought conditions, substance mineralization could be stimulated by P in the monoculture of *P. zhennan* and could improve the microbial community, but have no impact on the microbial biomass; (iii) the impact of P addition is weighty and possibly will improve the ability of soil microbes to resist drought. However, the effect of P under drought conditions is dependent on the planting system.

### 3.3. Nonadditive Effects of Species Mixture: Antagonistic and Synergistic

In this study, we found inconsistent patterns in the admixing effects as both additive and nonadditive effects appear to hinge upon which soil variable was being determined. Except in the drought with P addition treatment, the impact of admixing on total PLFAs, bacterial PLFA, and fungi PLFA was synergistic in all treatments. Therefore, this indicates that these communities could be maintained by the mixture of *A. cremastogyne* and *P. zhennan*, and that the complementary co-occurrence of these trees might offset whatever the inhibitory effects of environmental factors might be on the microbial community. Overall, the present findings indicate that, while mixing two monospecific tree species might exert positive effects on some soil variables, the impact of the mixture could be negative, or could be impossible to separate from their individual effects on other variables [7,32].

### 3.4. Linking Soil Enzyme Activities, Soil Chemical Variables, and PLFA Profiles

The correlations amongst enzyme, soil chemical properties, and PLFAs turned out to be uneven for all enzymes assayed and the planting system. In disparity to a previous study [11], DON and DOC in the mixed culture were negatively linked to the composition of microbial community, suggesting that feedback of microbial PLFAs in mixed cultures is indirect and probable due to treatment-induced disparities in soil variables [4]. The fact that urease was significantly higher and positively related to the microbial composition in MPA indicates its importance in enhancing N cycling [26]. Meanwhile, in the monoculture of *P. zhennan*, *β*G were positively related to the totPLFAs, total bacteria, AMF, G^+^, and G^−^ PLFAs, suggesting that *β*G activities are more useful for reflecting microbial metabolic activity under PZ than alkaline and urease activities. The variances in the effects of plants on community composition of soil organisms have mostly been attributed to species-specific characteristics, neglecting species mixtures [7,14]. Our findings, that the planting system had a differential effect on the pattern of links amongst the microbial community composition, soil chemical variables, and enzyme activities, confirmed the role of both individual plants and their additivity in determining the community compositions of microbes, and also suggest that there may be plant-mediated effects of other factors on soil microbial properties.

## 4. Materials and Methods

### 4.1. Experimental Setup

Top soil (pH of 7.3, total phosphorus (TP): 0.89 g/kg, available phosphorus (aP): 0.04 g/kg, total nitrogen (TN): 1.01 g/kg, total carbon (TC): 6.63 g/kg) was randomly collected from Dujiangyan Natural Forest at Sichuan, China. The region, which represents mild subtropical monsoon climate, has a temperature of 16.7 °C (mean annual) and precipitation of 945.6 mm. The litter and noticeable undecomposed materials in the topmost layer were removed. The soils were taken to the screen house at Chengdu Institute of Biology, China, and thoroughly homogenized to form a composite sample.

The experiment utilized a factorial design with three planting systems (monoculture of *P. zhennan* (PZ); monoculture of *A. cremastogyne* (AC); and a mixed culture (MPA)); water treatment (well-watered and drought); and P application (with and without P). To ensure enough plant materials, 90 pots (10 L) were filled with 8 kg soil samples and divided into three groups of 30 pots, with each group containing one of the three planting systems. After growing for two months to ensure proper establishment and the return of the saplings to normal growth, 20 well-grown saplings were selected from each group. Each group of twenty pots was split into four, such that every five pots (replicates) received a treatment of well-watered with P-addition (WPT); well-watered only (WT); drought with P-addition (DPT); or drought only (DT). The well-watered treatment was used as the control to monitor the effect of drought. Similarly, the well-watered with P treatment was used as the control for drought with P enrichment. Phosphorus was supplied as NaH_2_PO_4_ (25.5% P), at a proportion of 278.37 mg of P per pot, which represents an increment compared to previous studies. Before supplying water, we calculate the soil relative water content (SRWC) according to the weight technique of Xu et al. [33].

The SRWC was grouped into two levels: well-watered (80–85%) and drought (30–35%). Soil moisture was maintained at the respective target SRWC throughout the experiment. Twenty-four weeks after transplanting and supply of treatments, the soils were retrieved for the investigation of soil chemical and microbial parameters.

### 4.2. Analyses of Soil Chemical Characteristics

Soil nitrate (NO_3_^−^–N) was measured using flow injection autoanalyzer (AutoAnalyzer3, Bran Luebbe, Norderstedt, Germany) from the soil extract (with 50 mL of 2.0 M KCl). Dissolved organic carbon and nitrogen were removed from 10 g fresh soil sample with 2.0 M KCl at 20 °C and subsequently analyzed with TOC/TN analyzer (Multi N/C^®^ 2100(S), Analytik Jena AG, Jena, Germany). Moisture content of the soil was measured by mass lost after 10 g of moist soil was oven dried at 105 °C to a constant weight for 24 h. Molybdate-ascorbic acid method was used for the determination of available phosphorus (aP), after extracting the soil samples with 0.5 M NaHCO_3_ (pH 8.2) [34].

### 4.3. Microbial Biomass

The measurement of carbon, nitrogen, and phosphorus microbial biomass (mbc, mbn, and mbp, respectively) from fresh soil samples was carried out using chloroform fumigation–extraction protocol [35,36]. After 24 h in a vacuum desiccator, C, N, and P were removed from both the fumigated and unfumigated samples using 100 mL of 0.5 M NaHCO3 for P and 0.5 M K_2_SO_4_ for C and N. The extractions were measured using TOC/TN analyzer (Multi N/C^®^2100(S), Analytik Jena AG, Germany). Extracted phosphorus was measured calorimetrically using the technique of Olsen and Sommers [34]. The following formula was used for the calculation of the microbial biomass:

MB = E/k_E,_

where E is the difference between C, N, or P extracted from fumigated and nonfumigated soils, and k_E_ = 0.45 for MBC and MBN and 0.4 for MBP [37].

### 4.4. Analyses of Phospholipid Fatty Acids (PLFAs)

Phospholipid fatty acids were employed for characterizing structure of the microbial community. Using 8 g freeze-dried soil samples, phospholipids were extracted in a mixture of chloroform/methanol/phosphate (1:2:0.8, *v*/*v*/*v*) according to Bossio and Scow [38]. Both the saponification and transformation of the fatty acids in the chloroform phase into fatty acid methyl esters was carried out using strong acid methylation. PLFA methyl esters were detached and quantified by gas chromatography (6890N, Agilent, Santa Clara, CA, USA), attached to a flame ionization detector (19091B-102, Agilent, USA) and an HP-5 capillary column. Concentrations of each PLFA were computed following 19:0 internal standard concentration (nonadecanoic acid methyl ester). The information about the community structure was described using major microbial groups (abundance) with indicator fatty acid biomarker of 18:2ω6,9c, 16:1ω5c, 18:1ω9c, for fungal populations; a neutral lipid fatty acid (NLFA) biomarker of 16:1ω5c for arbuscular mycorrhiza fungi (AMF); i15:0, i16:0, a17:0, i14:0, a15:0, i17:0, i13:0, a16:0, i18:0 for Gram-positive bacteria (G^+^); and 16:1ω7c, 15:1ω6c, 16:1ω11c, 18:1ω7c, cy17:0, 17:1ω8c, 18:1ω5c for Gram-negative bacteria (G^−^). The totality of all microbial groups was used as the estimate of total microbial biomass (totPLFAs).

### 4.5. Determination of Enzyme Activities

The activities of three enzymes indicating N-cycling, C-cycling, and P-cycling (urease, β-glucosidase, and alkaline phosphatase) were tested using Tabatabai and Bremner [39] and Nannipieri et al. [40] approach. The activities of alkaline phosphatase were measured using p-nitrophenol phosphate (PNP). One gram of soil sample was mixed with 4 mL modified universal buffer (pH 6.5), 0.2 mL toluene, 1 mL substrate solution, and incubated for 1 h at 37 °C. To allow for color development, 1 ml of 0.5 mol/L NaOH and 4 mL of 0.5 mol/L CaCl_2_ was used to end the reaction. The soil solution obtained after filtering was measured colorimetrically at 410 nm. Activity of β-glucosidase was determined using 2 mL of MUB, pH 6, and 0.5 mL of 0.025 mol/L p-nitrophenyl β-D-glucopyranoside added to 1 g soil sample. The mixtures were incubated at 37 °C for 1 h and then determined colorimetrically at 405 nm. For the determination of urease activities, we incubated 2 g soil samples at 37 °C for 2 h with an aqueous urea solution. We removed the NH_4_^+^ released from the soil with a 1 mol/L KCl solution and measured it by a modified indophenol blue colorimetric reaction.

### 4.6. Data Analysis

Data obtained was verified for the assumptions of normality along with homogeneous variance using Shapiro–Wilk’s test. The tests revealed that the assumption of homogeneity was met for all the measured variables. We tested the differences in soil microbial biomass, microbial composition, enzyme activities, and soil chemical properties between controls (well-watered/well-watered with P addition) and the drought/drought with P addition conditions using independent-sample *t*-tests. All variables were further subjected to analysis of variance to examine the interplay among the three factors. The three factors were (i) planting systems (three levels), (ii) water treatments (two levels), and (iii) P treatments (two levels). The shifts in microbial community structure along the other selected soil variables in response to treatments under each planting system were assessed using principal component analysis (PCA). The relationships amongst the soil enzyme activities, soil chemical properties, and microbial PLFAs were determined using Spearman correlation coefficients. The principal component analysis was perform using “FactorMiner” package in R for windows (Version 3.4.4; R development Core Team 2017). We carried out other analysis using SPSS version 18.0 (SPSS Inc., Chicago, IL, USA). The additive or nonadditive aftermath of tree mixture on the selected microbial community, biomass, enzyme activities, and soil chemical properties were calculated for each treatment using

(1) $$\mathrm{NAE}\;=\frac{\left(O-E\right)}{E},$$

O represents the observed parameter response of the replicate of each treatment in the tree mixture, and E represents the expected response [40]. The expected responses (E) were determined using

$$E=\sum_{i=1}^{n}Ri/n,$$

where R*_i_* represents soil response when tree species *i* was added, and *n* is the overall number of species in the mixture. Values of (O–E)/E ratio differing significantly from zero after testing with *t*-test indicate nonadditive, whereas values that are not different significantly from zero designate additive mixing effects. Positive great values indicate synergistic mixing effects, whereas negative great values indicate antagonistic mixing effects.

## 5. Conclusions

This study demonstrates that under drought conditions, mineralization could be stimulated by the addition of increase level of P in the monoculture of *P. zhennan* and could meliorate the microbial community but have no leverage on the biomass. Drought decreased microbial N in all three planting systems. With the addition of P microbial biomass C and N, β-glucosidase activities and urease were higher in the mixture of *P. zhennan* and *A. cremastogyne.* This suggests that greater biological activity could be established with P addition under drought conditions in mixed planting systems. Overall, the sensitivity of microbial community to drought and P hinge on the organism group and is strongly shaped by the planting system.

## Figures and Tables

**Table 1:** Effect of treatments on the soil chemical properties under different planting systems.

Plant Types	Trt	NO_3_^−^–N (mg/g)	DON (mg/kg)	DOC (mg/kg)	SM (%)	aP (mg/kg)
Monoculture of *Phoebe* (PZ)						
	DT	2.8 ± 0.3 a	96.4 ± 0.4 a	253.2 ± 12.9 a	6.4 ± 1.0 b	89.9 ± 0.8 a
	WT	2.6 ± 0.1 b	93.8 ± 0.8 b	239.4 ± 3.7 a	9.4 ± 0.2 a	80.6 ± 0.7 b
						
	DPT	2.4 ± 0.0 b	107.4 ± 5.3 a	240.2 ± 3.5 a	6.9 ± 0.6 b	109.9 ± 4.3 a
	WPT	3.7 ± 0.2 a	98.7 ± 0.5 b	203.9 ± 6.5 b	8.3 ± 0.1 a	100.4 ± 1.7 b
Monoculture of *Alnus* (AC)						
	DT	2.8 ± 0.1 a	98.5 ± 2.1 a	156.3 ± 5.8 b	6.1 ± 0.3 b	77.9 ± 3.0 a
	WT	2.5 ± 0.1 b	92.3 ± 1.3 b	243.6 ± 1.5 a	9.4 ± 1.2 a	71.6 ± 2.2 b
						
	DPT	2.7 ± 0.1 a	96.8 ± 1.4 a	210.1 ± 8.3 b	7.2 ± 0.3 b	99.3 ± 2.3 a
	WPT	2.5 ± 0.0 b	95.7 ± 1.4 a	248.9 ± 2.9 a	9.4 ± 0.6 a	90.6 ± 3.3 b
Mixed culture (MPA)						
	DT	2.9 ± 0.1 a	100.3 ± 2.6 a	222.06 ± 5.9 a	5.6 ± 0.6 b	82.8 ± 3.0 b
	WT	3.3 ± 0.3 a	90.9 ± 0.9 b	191.7 ± 12.5 b	8.2 ± 0.4 a	93.0 ± 2.1 a
						
	DPT	2.7 ± 0.3 a	103.4 ± 1.9 a	249.0 ± 5.1 b	6.3 ± 0.6 b	111.9 ± 3.8 a
	WPT	3.2 ± 0.2 a	99.7 ± 2.3 a	342.3 ± 26.0 a	9.1 ± 1.4 a	106.1 ± 2.1 b

Each value is the mean ± SE (*n* = 5). Dissimilar lower-case letters indicate significant differences among the treatments at *p* < 0.05. PZ: *Phoebe zhennan;* AC: *Alnus cremastogyne*; MPA: mixed culture of *Phoebe zhennan* and *Alnus cremastogyne*. Trt: treatments; DT: drought; WT: optimum water; DPT: drought plus phosphorus addition; WPT: optimum water plus phosphorus addition. SM: soil moisture (%); DOC: dissolved organic carbon (mg/kg); DON: dissolved organic nitrogen (mg/kg); NO_3_^−^–N: soil nitrate nitrogen(mg/g); aP; available phosphorus (mg P kg^−1^ soil).

**Table 2:** Impact of the treatments on the soil enzymes activities under different planting systems.

Plant Types	Trt	Alkaline Phosphatase (AP) (mg p-NP kg^−1^h^1^)	*β-*Glucosidase (BG) (mg p-NP kg^−1^h^−1^)	Urease (mg NH_4_^+^–N kg^−1^ h^−1^)
Monoculture of *Phoebe* (PZ)				
	DT	50.8 ± 5.4 b	157.4 ± 14.7 b	40.4 ± 0.3 a
	WT	67.6 ± 7.9 a	184.1 ± 6.0 a	34.02 ± 1.3 b
	DPT	60.9 ± 6.9 a	174.3 ± 17.6 a	47.3 ± 0.9 a
	WPT	52.9 ± 1.0 b	149.9 ± 4.3 b	33.3 ± 2.1 b
Monoculture of *Alnus* (AC)				
	DT	88.2 ± 0.3 b	144.4 ± 14.5 b	36.79 ± 1.5 a
	WT	104.8 ± 12 a	198.7 ± 5.9 a	31.8 ± 0.2 b
	DPT	124.1 ± 3.1 a	145.9 ± 2.7 b	42.8 ± 0.3 a
	WPT	131.0 ± 5.9 a	243.9 ± 37.0 a	42.6 ± 0.4 a
Mixed culture (MPA)				
	DT	102.3 ± 4.3 a	171.1 ± 9.4 a	35.8 ± 1.3 b
	WT	30.4 ± 4.2 b	196.4 ± 14.7 a	49.8 ± 2.9 a
	DPT	104.3 ± 3.4 b	216.1 ± 3.5 a	42.7 ± 2.2 a
	WPT	152.5 ± 16.9 a	169.2 ± 12.9 b	36.1 ± 0.3 b

Each value is the mean ± SE (*n* = 5). Dissimilar lower-case letters indicate significant differences among the treatments at *p* < 0.05. PZ: *Phoebe zhennan;* AC: *Alnus cremastogyne*; MPA: mixed culture of *Phoebe zhennan* and *Alnus cremastogyne*. Trt: treatments; DT: drought; WT: optimum water, DPT: drought plus phosphorus addition; WPT: optimum water plus phosphorus addition.

## Data Availability

The data presented in this study are available in all Tables and Figures of the manuscript.

## Supplementary Materials

The following supporting information can be downloaded at: https://www.mdpi.com/article/10.3390/plants11030319/s1, Table S1: The interactive effects of planting systems, phosphorus (P) addition and water treatment on the soil chemical properties variables; Table S2: Summary of three-way ANOVA investigating the effects of planting systems, water treatments, phosphorus addition and their interaction on soil microbial biomass; Table S3: Effects of plant systems, water treatments, phosphorus addition and their interaction on microbial community; Table S4: Summary of three-way ANOVA investigating the effects of planting systems, water treatments, phosphorus addition and their interaction on soil enzymes activities.
