# Supplementary material for: Planting Systems Affect Soil Microbial Communities and Enzymes Activities Differentially under Drought and Phosphorus Addition
Source: Plants (Basel). 2022 Jan 25;11(3):319. doi: 10.3390/plants11030319 (PMC8839870; doi:10.3390/plants11030319)
Supplement: Supplementary file 1 [file plants-11-00319-s001.zip › plants-1549976-supplementary.pdf]

Planting systems affects soil microbial communities and enzymes activities differentially under drought and phosphorus addition

Olusanya Abiodun OLATUNJI [1,2*], Kaiwen PAN [2**], Akash TARIQ[2,3], Gideon Olarewaju OKUNLOLA[1], Dong WANG [4], Idris Olawale RAIMI[5], Lin ZHANG[2]

[1]Department of Plant Biology, Faculty of Basic and Applied Sciences, Osun State University, Osogbo, Nigeria.

[2]CAS Key Laboratory of Mountain Ecological Restoration and Bioresource Utilization & Ecological Restoration Biodiversity Conservation Key Laboratory of Sichuan Province, Chengdu Institute of Biology, Chinese Academy of Sciences, Chengdu 610041, People's Republic of China.

[3] Xinjiang Key Laboratory of Desert Plant Roots Ecology and Vegetation Restoration, Xinjiang Institute of Ecology and Geography, Chinese Academy of Sciences, Urumqi 830011, China.

[4] College of Environmental Science and Engineering, China West Normal University, Nanchong 637000, China

[5] Department of Biology, Sefako Makgatho Health Sciences University, Pretoria, South Africa.

* Corresponding author

E-mail address: *olusanya084@yahoo.ca, ** pankw@cib.ac.cn (K. Pan)

Table S1: The interactive effects of planting systems, phosphorus (P) addition and water treatment on the soil chemical properties variables.

Effects	NO_3^--N		DON		DOC		SM		aP	
	F	P	F	P	F	P	F	P	F	P
PS	6.7	**0.003**	2.4	0.10	12.5	**<0.001**	0.49	0.61	30.4	**<0.001**
WT_t	3.2	0.07	8.0	**0.007**	15.1	**<0.001**	31.8	**<0.001**	0.12	0.72
PT_t	0.04	0.83	0.9	0.34	7.1	**0.02**	0.08	0.77	161.6	**<0.001**
PS *WT_t	1.9	0.14	7.1	**0.002**	18.6	**<0.001**	0.19	0.82	7.6	**0.001**
PS * PT_t	0.9	0.37	8.7	**0.001**	38.0	**<0.001**	0.62	0.53	8.51	**0.02**
WT_t * PT_t	0.1	0.97	4.3	**0.04**	11.9	**0.001**	2.4	0.12	1.9	0.16
PS * WT_t *PTt	2.1	0.13	0.9	0.38	17.4	**<0.001**	0.95	0.39	4.1	**0.02**

Statistically significant ($p < 0.05$) results are shown in boldface (n = 5). PS: planting systems; WT_t: Water treatments and PT_t: Phosphorus addition. SM: soil moisture (%); DOC: dissolved organic carbon (mg/kg); DON: dissolved organic nitrogen (mg/kg); NO_3^--N: soil nitrate nitrogen(mg/g) and aP: Available phosphorus (mg P kg^{-1}soil).

Table S2: Summary of three-way ANOVA investigating the effects of planting systems, water treatments, phosphorus addition and their interaction on soil microbial biomass

Effects	MBP		MBN		MBC	
	F	P	F	P	F	P
**PS**	3.02	**0.05**	11.8	**<0.001**	8.8	**0.001**
**WT_t**	13.9	**0.001**	0.02	0.90	14.8	**<0.001**
**PT_t**	18.7	**<0.001**	0.26	0.61	9.7	**0.003**
**PS *WTs**	14.1	**<0.001**	0.79	0.45	0.93	0.39
**PS * PT_t**	33.5	**<0.001**	6.0	**0.005**	18.8	**<0.001**
**WT_t * PT_t**	23.9	**<0.001**	22.2	**<0.001**	0.23	0.63
**PS * WT_t *PT_t**	16.1	**<0.001**	3.4	**0.04**	5.6	**0.006**

Statistically significant ($p < 0.05$) results are shown in boldface (n = 5). PS: planting systems; WT_t: Water treatments and PT_t: Phosphorus addition. MBP: microbial biomass phosphorus; MBN: microbial biomass nitrogen and MBC: microbial biomass carbon.

Table S3: Effects of plant systems, water treatments, phosphorus addition and their interaction on microbial community

Parameters		(PS)	(WT_t)	(PT_t)	PS * WT_t	PS * PT_t	WT_t *PT_t	PS* WT_t* PT_t
Total PFLAs	*F*	53.9	537.6	289.7	35.2	116.1	1297.3	75.0
	P	**<0.001**	**<0.001**	**<0.0001**	**<0.0001**	**<0.0001**	**<0.0001**	**<0.0001**
Bacteria PFLAs	*F*	52.4	635.5	210.2	44.4	112.2	1037.8	83.3
	P	**<0.0001**	**<0.0001**	**<0.0001**	**<0.0001**	**<0.0001**	**<0.0001**	**<0.0001**
G^- bacteria	*F*	52.1	248.1	53.3	42.6	73.7	520.3	49.4
	P	**<0.0001**	**<0.0001**	**<0.0001**	**<0.0001**	**<0.0001**	**<0.0001**	**<0.0001**
G^+ bacteria	*F*	4.8	425.3	206.0	4.6	34.3	508.1	31.0
	P	**0.011**	**<0.0001**	**<0.0001**	**0.01**	**<0.0001**	**<0.0001**	**<0.0001**
Fungi PFLAs	*F*	20.3	71.7	63.5	1.10	23.6	267.6	9.5
	P	**<0.0001**	**<0.0001**	**<0.0001**	0.34	**<0.0001**	**<0.0001**	**<0.001**
AMF (**16:1ω5c**)	*F*	3.8	101.0	66.3	0.7	20.5	217.2	9.4
	P	**0.02**	**<0.0001**	**<0.0001**	0.49	**<0.0001**	**<0.0001**	**<0.001**
F: B ratio	*F*	3.6	17.2	5.5	1.7	4.1	4.3	3.5
	P	0.3	0.1	0.2	0.35	0.2	0.4	0.3

Significant *P*-values ($p < 0.05$) shown in bold face type. PT_s: planting systems; WT_t: Water treatments and PT_t: Phosphorus addition.

Table S4: Summary of three-way ANOVA investigating the effects of planting systems, water treatments, phosphorus addition and their interaction on soil enzymes activities

Effects	Alkaline phosphate (ug.g-1.h-1)		β-Glucosidase (mg/kg/hr)		urease (μg N-NH_4 g^{-1} hr^{-1})	
	F	*P*	F	*P*	F	*P*
PS	49.4	**<0.001**	2.3	0.11	3.9	**0.02**
WT_t	1.6	0.20	6.6	**0.01**	13.6	**0.001**
PT_t	38.3	**<0.001**	0.83	0.36	10.9	**0.002**
PS *WT_t	4.8	**0.01**	9.9	**<0.001**	23.7	**<0.001**
PS * PT_t	15.1	**<0.001**	1.1	0.32	17.2	**<0.001**
WTs * PT_t	11.1	**0.002**	2.3	0.13	22.3	**<0.001**
PS * WT_t *PT_t	26.7	**<0.001**	4.2	**0.02**	19.7	**<0.001**

Statistically significant ($p < 0.05$) results are shown in boldface (n = 5). PS: planting systems; W_t: Water treatments and P_t: Phosphorus addition.
